# Understanding the mechanisms of mitochondrial rewiring during viral infections

**DOI:** 10.1099/jgv.0.002128

**Published:** 2025-07-07

**Authors:** Marta Lopez-Nieto, Nicolas Locker

**Affiliations:** 1 Faculty of Health and Medical Sciences, School of Biosciences and Medicine, University of Surrey, Guildford, UK; 2 The Pirbright Institute, Pirbright, UK

**Keywords:** innate immunity, mitochondria, mitochondrial unfolded protein response (UPR^mt^)

## Abstract

As intracellular parasites, viruses must hijack and often rewire organelles, signalling pathways and the bioenergetics machinery of the infected cell to replicate their genome, produce viral proteins and assemble new viral particles. Mitochondria are key eukaryotic organelles often referred to as the cell’s powerhouse. They control many fundamental cellular processes, from metabolism and energy production to calcium homeostasis and programmed cell death. Importantly, mitochondrial membranes are also critical sites for the integration and amplification of antiviral innate immune responses. Overall, mitochondria are therefore both supporting the virus life cycle by sustaining energy production, metabolism and synthesis of macromolecules and part of the cell’s first line of defence against viruses. This review summarizes recent findings on viral manipulations of mitochondria and their functions. We explore the evolving understanding of how mitochondrial dynamics is targeted to regulate innate immunity, evasion strategies used to avoid mitochondrial-associated mechanisms that impair replication and the role of mitochondrial functions such as generating reactive oxygen species or regulating the electron transport chain during infection. Overall, we provide a comprehensive view of how viruses modulate mitochondrial function to promote replication.

## Introduction

Mitochondria are membrane-bound organelles, present in eukaryotic cells whose origin is considered to have occurred more than two billion years ago from an ancient symbiosis between a proto-eukaryotic cell (host) and an alpha proteobacterium (procaryote) [1]. Unlike other organelles, mitochondria are surrounded by two membranes: an outer mitochondrial membrane (OMM) separates the mitochondrion from the cytoplasm and an inner mitochondrial membrane (IMM) delimits the mitochondrial lumen (matrix) [2]. The IMM contains the assembled complexes and subunits of the electron transport chain (ETC) and ATP synthase and, therefore, is the main site of energy production within the cell [3]. The OMM is important for organelle physiology as it hosts proteins involved in the import of mitochondrial protein precursors, contact with other organelles, signalling cascades as well as most of the proteins involved in mitochondrial dynamics [4].

Mitochondria are highly dynamic and continuously changing their shape, network complexity and distribution within cells in response to cellular and environmental conditions. One of the main functions of mitochondria is the production of energy, in the form of ATP, through oxidative phosphorylation (OXPHOS). Electrons are transported by ETC complexes while protons are pumped into the intermembrane space, where they accumulate and produce an electrochemical gradient known as mitochondrial membrane potential (MMP). Protons take advantage of this electrochemical gradient to move back into the mitochondrial matrix through complex V, also known as ATP synthase, to generate ATP [5]. Electron leakage from the ETC complexes, during transport between complexes, typically occurs at the sites of semiquinone radical or a reduced flavin and can result in the production of mitochondrial reactive oxygen species (mtROS) and oxidative stress [6]. In addition to energy conversion and metabolic processes, mitochondria are considered cell signalling hubs, as they are implicated in the regulation of cell death, innate immunity and cell differentiation, amongst others [7,8].

As obligate parasites, viruses exploit host cellular machinery to replicate their genome, produce viral proteins and assemble virion progeny. Viral infection causes dramatic rearrangements in cell architecture, alters metabolism and modulates the physiology of various organelles [9]. Given the multiple roles of mitochondria, viruses have evolved strategies to hijack mitochondrial dynamics and functions to favour their infection. Accordingly, multiple viruses encode proteins that directly or indirectly target mitochondria and modulate mitochondrial dynamics and functions for their own benefit. Here, we review how viruses manipulate mitochondrial dynamics, mitophagy, redox homeostasis, host antiviral responses and mitochondrial apoptotic pathways. Regulation of bioenergetics and metabolism during infection is less well understood and will be outside the focus of this review [10].

## Regulation of mitochondrial dynamics and mitophagy by viruses

Mitochondrial morphology is determined by a balance between fusion and fission events [11]. Mitochondrial fission, defined as mitochondrial fragmentation, relies on dynamic-related protein 1 (Drp1) [12]. Upon activation, Drp1 is recruited from the cytosol to mitochondria, particularly at the mitochondrial–ER contact sites [13], and binds to its OMM receptors, including mitochondrial fission factor, mitochondrial dynamics proteins of 49 and 51 kDa (MiD49 and MiD51, respectively) and mitochondrial fission 1 protein (Fis1) [14,16]. Drp1 recruitment and activity are regulated by two major phosphorylation sites on Drp1, Ser616 and Ser637, each regulating Drp1 fission activity but in opposite manners [17]. Drp1 Ser616 phosphorylation promotes its translocation to the mitochondria and its subsequent fission activity, while phosphorylation of Drp1 at Ser637 decreases its GTPase activity and retains Drp1 in the cytosol, blocking fission and, in turn, resulting in mitochondrial elongation [18,19]. Mitochondrial fusion is mediated by the GTPases mitofusin 1 and 2 (Mfn1 and Mfn2), found in the OM, and optic atrophy protein 1 (OPA1), located in the IMM [20,21]. Growing evidence suggests that regulation of mitochondrial dynamics is essential to maintain optimal mitochondrial integrity and, thereby, cellular function. For instance, cellular adaptation to metabolic conditions involves remodelling of mitochondrial structure [22,24]. Furthermore, mitochondrial fragmentation is required for the correct removal of damaged mitochondria through mitophagy [25].

**Fig. 1.**
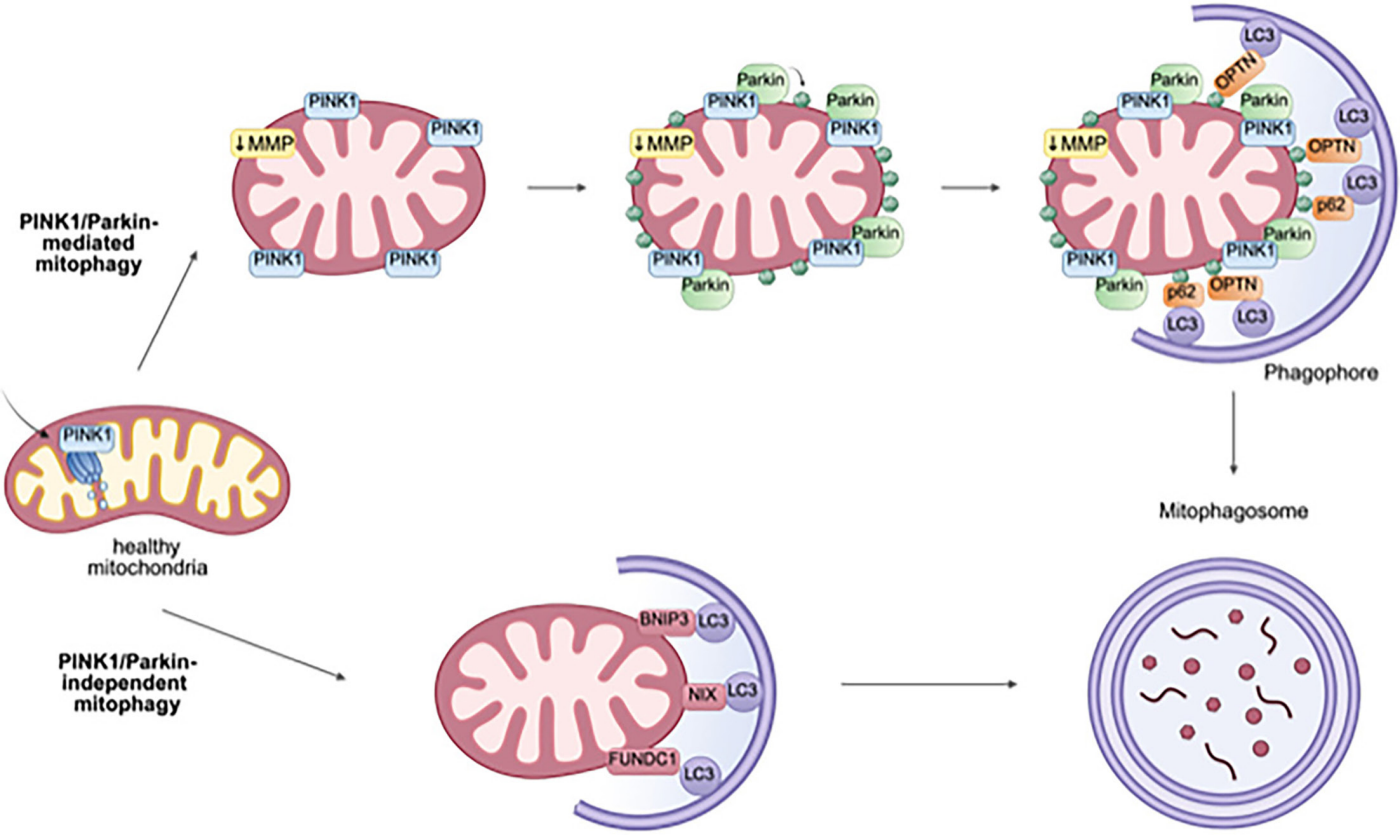
Molecular mechanisms of mitophagy. In healthy mitochondria, PINK1 is imported into mitochondria where it is cleaved and degraded. Disruption of MMP in stressed mitochondria impairs PINK1 import, resulting in accumulation of PINK1 on the OM. PINK1 recruits and activates Parkin, which ubiquitinates OM proteins. Poly-ubiquitinated proteins are recognized by protein adaptors such as p62 and OPTN, which further recruit phagophore to damaged mitochondria by interacting with LC3, resulting in mitophagosome formation. Alternatively, OM proteins including BNIP3, NIX and FUNDC1 can act as receptors and directly interact with LC3, leading to mitophagosome formation. Mitophagosomes are subsequently fused with lysosomes for further degradation. Figure created with BioRender.

Mitophagy is the selective degradation of severely dysfunctional mitochondria through the autophagy-lysosomal pathway, where damaged mitochondria are sequestered within mitophagosomes and further translocated to lysosomes for degradation, a normal repair process central to maintain homeostasis under basal conditions [26]. The two primary pathways that regulate mitophagy include (a) the canonical PTEN-induced putative kinase 1 (PINK1)/Parkin system [27] and (b) mitophagy receptors at the OMM (Fig. 1). In healthy mitochondria, PINK1 is imported into mitochondria where it is cleaved and degraded [28]. Disruption of MMP in stressed mitochondria impairs PINK1 import, resulting in accumulation of PINK1 on the OMM. PINK1 recruits and activates the E3 ubiquitin ligase Parkin, which subsequently ubiquitinates OMM proteins [29]. Poly-ubiquitinated proteins are recognized by protein adaptors such as p62 and optineurin (OPTN), which further recruit phagophore to damaged mitochondria by interacting with LC3, resulting in mitophagosome formation [30]. Mitophagy can also be induced by PINK1-independent pathways where OMM proteins, including pro-apoptotic B cell lymphoma 2 (BCL-2) interacting protein 3 (BNIP3), NIP3-like protein X (NIX) and FUN14 domain-containing protein 1 (FUNDC1), which act as mitophagy receptors and directly interact with LC3, lead to mitophagosome formation and mitochondrial elimination [31]. The exact reasons behind the selection of a specific mitophagy pathway by cells remain to be elucidated.

Zika virus (ZIKV), a positive ssRNA virus belonging to the *Flaviviridae* family, induces mitochondrial fragmentation in different cell types, including A549 cells [32], human retinal cells [33] and placental JEG-3 cells [34], as well as neuronal cell lines [35], which is associated with a loss in MMP and increased reactive oxygen species (ROS) levels. Specifically, ZIKV non-structural protein 4A (NS4A) promotes Drp1 phosphorylation at Ser616, which is responsible for mitochondria fission in JEG-3 cells [34]. Moreover, ZIKV infection reduces levels of the pro-fusion proteins Mfn2 and OPA1 in neuronal cells [35]. Consequently, Drp1 silencing restores ZIKV-induced mitochondrial fragmentation and results in reduced viral RNA levels in placental JEG-3 cells, while aggravation of mitochondrial fission by Mfn2 silencing increases viral replication, demonstrating that mitochondrial fragmentation has a pro-viral role for ZIKV replication, which could be due to the downregulation of antiviral signalling occurring at the mitochondrial membrane. Mitochondrial fragmentation is one of the first required events for clearance of damaged mitochondria via mitophagy, and given the ZIKV-induced mitochondrial fission, one could expect that ZIKV also promotes mitophagy. However, studies have found conflicting results regarding how ZIKV modulates mitophagy and its effect on viral replication. On one hand, niclosamide-induced mitophagy reduced ZIKV viral titres in both A549 cells and mice and relieved brain necrosis in mice models, while reduced mitophagy by PINK1 knockdown promoted ZIKV infection in A549 infected cells [32], indicating that mitophagy has an antiviral effect. Similarly, Ponia *et al.* showed that ZIKV NS5 binds to a host factor called Ajuba, which promotes mitophagy in HEK293T cells by enhancing PINK1 autophosphorylation [36]. This binding prevents the translocation of Ajuba to the mitochondria and, in turn, mitophagy. ZIKV NS5-mediated mitophagy suppression does not influence viral replication but enhances viral dissemination in mice by amplifying pro-inflammatory chemokine production, potentially through mtRNA-dependent PKR activation. In contrast to these results, Lee *et al*. found that ZIKV infection with different strains triggers mitophagy in human trophoblast and neuronal progenitor cells [34]. ZIKV NS4A ectopic expression results in depolarization of MMP and is sufficient to cause mitophagy, suggesting that NS4A drives ZIKV-induced mitophagy in placental JEG-3 cells. Moreover, ZIKV promotes both PINK1/Parkin-dependent and -independent pathways, as shown by increased protein levels of PINK1 and the mitophagy receptors BNIP3 and NIX during viral infection [34]. Mitophagy suppression by PINK1, BNIP3 and NIX silencing reduces ZIKV viral replication in JEG-3 placental cells, highlighting the pro-viral role of mitophagy. The discrepancies between the aforementioned studies could be explained due to the use of different viral strains, cell types and viral proteins studied, as well as the techniques employed to detect mitophagy. In summary, ZIKV infection induces mitochondrial fragmentation and modulates mitophagy; however, further studies are required to clarify whether these differences determine strain pathogenicity or neurological symptoms, as well as whether mitophagy benefits the virus or the host cell.

Japanese encephalitis virus (JEV) infection, another enveloped ssRNA virus belonging to the *Flaviviridae* family, also promotes mitochondrial fragmentation in Huh7 cells. Accordingly, reduced levels of pro-fusion proteins, including Mfn1 and Mfn2, and increased Drp1 Ser616 phosphorylation are observed during JEV infection [37]. Moreover, NS4A expression, which localizes to mitochondria, is sufficient to recapitulate this mitochondrial phenotype as well as to trigger mitophagy by interacting with PINK1. Similarly to ZIKV, Drp1 and PINK1 silencing disrupts JEV-induced mitochondrial fission and mitophagy, respectively, and significantly reduces viral replication in Huh7 cells, suggesting that mitophagy is advantageous for JEV fitness. Moreover, another study observed lower mtDNA copy numbers in the cortex, brain stem and hippocampus of JEV-infected mice [38], indicating a decrease in mitochondria which is consistent with enhanced mitophagy, despite decreased mRNA levels of fission proteins, such as Drp1 and Fis1, and upregulation of Mfns and OPA1 mRNA levels compared to control. These proteins are modulated at the translation and post-translation level rather than the transcription level; therefore, these results are not enough to suggest that fission/fusion mitochondrial balance was shifted towards fusion since neither protein levels of mitochondrial dynamics machinery nor mitochondria morphology in JEV-infected mice cells are shown. Altogether, this evidence indicates that JEV promotes mitochondrial fragmentation and fusion to facilitate replication; however, the mechanisms underlying this process need further investigation. In addition, while ZIKV mainly circulates between mosquito vectors and humans, JEV has a broader host range, including insect vectors, swine and avian species, with humans as a dead-end host. Thus, it would be interesting to understand how these differences in mitochondrial regulation may impact the transmission of JEV and ZIKV and host range.

In contrast to ZIKV and JEV, conflicting evidence has been reported regarding the modulation of mitochondrial dynamics during dengue virus (DENV) infection. Two independent reports found that mitochondria undergo elongation upon DENV in Huh7 cells. Specifically, Barbier *et al.* reported the association of DENV NS4B and NS3 proteins with mitochondria as well as the reduction of mitochondrial pro-fission proteins including Mfn1, Mfn2 and Drp1 in DENV-infected Huh7 cells [39]. Moreover, induction of mitochondrial fission by CCCP treatment or Mfn2 silencing decreases DENV replication. These findings are supported by Chatel-Chaix *et al.*’s prior demonstration that DENV infection induces mitochondria elongation and alters mitochondria–ER contacts in Huh7 cells [40]. In particular, DENV NS4B drives mitochondrial elongation by inhibiting Drp1 mitochondrial translocation and subsequent activation. Moreover, it was shown that, by silencing Drp1 or Mfn2, mitochondrial fusion enhances DENV replication while suppressing DENV-induced IFN response. Furthermore, Molino *et al.* developed a compound called Mito-C that targets nutrient-deprivation autophagy factor 1 from NEEFT protein family, an important regulator of mitochondrial dynamics, facilitating Drp1 recruitment to the ER–mitochondrial contact sites and promoting mitochondrial fission [41]. Treatment with Mito-C abolishes DENV-induced mitochondrial fragmentation and restores ER–mitochondria contacts in Huh7 cells, leading to a reduction of DENV replication. Taken together, these results indicate that DENV shifts the balance of fission/fusion towards fusion, promoting mitochondria elongation, which is crucial for its replication. In contrast to these studies, Yu *et al.* found that levels of fission-related proteins, including Drp1, p-Drp1 and OPA1, remain unchanged during DENV infection in A549 cells, while the fusion Mfn1 and Mfn2 proteins are significantly reduced, suggesting that DENV negatively regulates fusion [42]. Specifically, it was shown that DENV protease NS2B3 cleaves Mfns and its expression is sufficient to promote fragmented mitochondria. Moreover, DENV infection of the A549 cell line overexpressing Mfn1 results in higher IFN production and lower viral titres, suggesting that DENV infection promotes mitochondrial fission to dampen antiviral signalling and facilitate viral infection. In agreement with these findings, Singh *et al.* reported partial mitochondrial fragmentation in Huh7 cells upon DENV infection with all serotypes (DENV1–DENV4), although with serotype-specific differences in the degree and rate of fission [43]. However, the mechanism by which DENV induces fragmentation remains unknown given the observed reduced levels of both pro-fission proteins, including Drp1 Ser616 phosphorylation, and pro-fusion proteins upon DENV infection. Intriguingly, DENV infection also inhibits Parkin-mediated mitophagy, leading to mtDNA release from damaged mitochondria and subsequent inflammasome activation.

Overall, the state of mitochondrial fusion/fission during DENV remains unclear given the above-mentioned conflicting reports. Several matters, including basal state of the cells, virus-dose inoculation and duration of the infection, as well as experimental approach, should be considered to explain the divergent published data. For instance, non-infected cells from Singh *et al.* already presented a highly elongated mitochondrial network, while a normal tubular network was observed in non-infected cells from both Barbier *et al.* and Chatel-Chaix *et al*., suggesting that the basal status of the cells as well as the basal levels of the proteins involved in fission and fusion machinery could explain the different results. Moreover, both Barbier *et al.* and Chatel-Chaix *et al*. observed mitochondrial elongation by staining mitochondria in DENV-infected cells, while Yu *et al*. used a heterokaryon formation assay, a more artificial approach, to suggest that DENV alters mitochondrial dynamics, without directly visualizing mitochondrial morphology in DENV-infected cells. Therefore, while these studies indicate that DENV modulates mitochondrial dynamics and functions to facilitate its replication, more studies are needed to disentangle the exact underpinning mechanisms.

Little is known about the regulation of mitochondrial dynamics during yellow fever virus (YFV) infection, an ssRNA virus from the *Flaviviridae* family. A recent preprint reported mitochondrial fragmentation upon YFV infection with the vaccine strain 17D at 24 h post-infection in HepG2 cells [44]. However, induction of mitochondrial elongation by chemically inhibiting Drp1 does not affect viral replication, while CCCP-induced mitochondrial fragmentation decreases viral titres. Thus, it is unclear how the modulation of mitochondrial dynamics by YFV impacts its replication, and further work is required.

Hepatitis C virus (HCV), an ssRNA virus from the *Flaviviridae* family, promotes mitochondrial fragmentation by inducing Drp1 phosphorylation at Ser616 in Huh7 cells and subsequent Parkin-mediated mitophagy [45,46]. Kim *et al.* showed that disruption of HCV-induced mitochondrial fission and mitophagy by Drp1 and Parkin silencing, respectively, results in a reduction in viral replication and an increase in antiviral signalling. More recently, Jassey *et al.* revealed that NS5A plays an important role in regulating mitophagy. Specifically, NS5A promotes mitochondrial fragmentation, loss of MMP and Parkin mitochondrial translocation in Huh7 cells [47]. Interestingly, HCV core protein interacts with Parkin and suppresses mitophagy by blocking Parkin mitochondrial translocation [48]. Therefore, all these findings demonstrate that HCV induces mitochondrial fission followed by mitophagy, facilitating viral replication. The distinct pro- and anti-mitophagy effects of specific HCV viral proteins highlight a more complex and spatiotemporal regulation of virus-induced mitophagy, which could contribute to temporal regulation of antiviral signalling.

Hepatitis B virus (HBV) is a partially dsDNA and circular virus belonging to the *Hepatoviridae* family, which induces Drp1-mediated mitochondrial fragmentation in Huh7 cells followed by Parkin-dependent mitophagy [49]. HBV induces Drp1 phosphorylation at Ser616 and its mitochondrial translocation, promoting mitochondrial fission. In addition, mitochondrial recruitment of Parkin is triggered in HBV-infected cells, driving mitophagy. Several studies have reported the mitochondrial localization of HBV HBx protein [49,51]. HBx promotes mitochondrial translocation of Parkin by upregulating PINK1 [49] as well as MMP disruption and ROS production [50]. Consequently, degradation of mitochondrial HBx by a mitochondrial E3 ubiquitin ligase, MARCH5, results in suppression of ROS production, mitophagy and hepatic inflammation [52]. Thus, HBV-induced mitochondrial fragmentation and mitophagy favour replication, although the exact mechanisms underlying these effects are not fully understood.

PB1-F2 protein from influenza A virus (IAV), an enveloped RNA virus from the *Orthomyxoviridae* family, localizes into the mitochondrial inner space through the TOM40 channel. PB1-F2 mitochondrial accumulation in HEK293T cells induces loss of MMP, which triggers OPA-1 processing and the subsequent Drp1 recruitment, resulting in mitochondrial fragmentation via Drp1 [53]. PB1-F2 induces mitophagy by interacting with mitochondrial Tu translation elongation factor through its C-terminal domain and acting as a mitophagy receptor through LC3 binding [54]. Interestingly, it was also demonstrated that influenza infection triggers Ulk1- and BNIP3-dependent mitophagy in A549 cells, which is essential for its viral replication [55]. Highly pathogenic influenza strains (such as the H5N1 subtype) express PB1-F2 in full length. In contrast, low pathogenic influenza strains (such as H1N1, except the 1918 pandemic strain) encode a shorter PB1-F2 protein that lacks the C-terminal domain, where the mitochondrial translocation signal is found [56]. Consequently, this short PB1-F2 isoform does not localize to mitochondria nor affect mitochondrial functions [53,57]. Furthermore, IAV nucleoprotein also localizes to mitochondria and induces mitophagy, which facilitates viral replication and enhances virus pathogenicity by inhibiting innate immunity in HEK293T cells [58]. These results suggest that IAV has evolved different strategies to induce mitochondrial fragmentation and mitophagy to enhance viral replication. In contrast, another study reported enhanced fusion of mitochondria upon H1N1 IAV infection in A549 cells as well as disruption of mitochondria–ER contact sites [59]. H1N1 IAV infection alters mitochondrial dynamics by decreasing Drp1 mitochondrial recruitment and increasing OPA1 expression. In addition, Mito-C-induced mitochondrial fission abolishes mitochondrial elongation induced by IAV, restores mitochondrial–ER contact sites and inhibits viral replication by potentiating RIG-I signalling. These discrepancies can be explained by the use of viral strains with different pathogenicity. Full-length form of PB1-F2 acts as a pro-fission factor and it is encoded by high pathogenicity strains. However, the H1N1 IAV variant which is associated with mitochondria elongation does not express the PB1-F2 full version. Future studies should aim to investigate whether the differences in mitochondrial dynamics regulation are associated with influenza pathogenicity.

Severe acute respiratory syndrome coronavirus (SARS-CoV) is an ssRNA virus that belongs to the Betacoronavirus genus. SARS-CoV ORF9b localizes to mitochondria in A549 cells and promotes mitochondrial elongation by inducing proteasomal degradation of Drp1 and triggers the degradation of mitochondrial antiviral signalling protein (MAVS), a key adapter protein in the amplification of IFN signalling, resulting in suppression of antiviral signalling [60]. These results demonstrate that SARS-CoV ORF9b manipulates mitochondrial dynamics and interferes with the antiviral immune response to promote viral replication. SARS-CoV-2 belongs to the same family as SARS-CoV and caused a global pandemic in 2020–2023 [61]. SARS-CoV-2 infection also results in loss of MMP, which leads to mtROS release and mitochondrial dysfunction in both Huh7 and Vero cells. PINK1/Parkin-mediated mitophagy is activated by the host cells to remove damaged mitochondria and maintain mitochondrial homeostasis; however, SARS-CoV-2 inhibits p62 and LC3 interactions, blocking engulfment of p62-label mitochondria into lysosomes and thereby suppressing mitochondrial clearance [62]. Moreover, ORF9b modulates mitochondrial biogenesis by impairing translocase outer membrane 70 (TOM70) mediated mitochondrial protein import, thus reducing mitochondrial proteins and mitochondrial volume [63]. Interestingly, a recent study has shown that SARS-CoV-2 infection induces abnormal mitochondrial elongation in HEK293T cells, which is associated with an increase in MMP and OXPHOS during early stages of infection. SARS-CoV-2-mediated mitochondrial modulation increases cell survival by activating the epidermal growth factor receptors signalling cascade, ensuring robust viral propagation [64].

Overall, these studies indicate that some viruses, including ZIKV, JEV, HCV, HBV and IAV, all induce mitochondrial fragmentation followed by complete mitophagy, which is important to maintain mitochondrial homeostasis during infection and favour viral replication by potentially dampening antiviral innate response and/or suppressing the mitochondrial intrinsic pathway (discussed below). In contrast, other viruses such as DENV, IAV (low pathogenicity strain, H1N1), SARS-CoV and SARS-CoV-2 promote mitochondrial fusion which facilitates viral replication by either disrupting innate immunity or aberrantly elevating mitochondrial bioenergetics (Fig. 1). It is surprising that viruses would promote mitochondrial elongation since it has been associated with enhanced antiviral signalling [65]. However, these discrepancies may be explained by the fact that virus-induced aberrant mitochondrial fusion also disrupts ER–mitochondria contacts which play an essential role in MAVS aggregation and subsequent IFN and cytokine production [40,66, 67].

## Role of mitochondria as antiviral signalling platform during infection

Mitochondria function as platforms for antiviral immune signal transduction due to the presence of MAVS in the OM, which is required for proper innate signalling [68]. RIG-I and melanoma differentiation-associated antigen 5 (MDA5) sense and detect dsRNA via their DExD/H box RNA helicase domain [69]. Upon dsRNA binding, both receptors undergo conformational changes which promote their dimerization and subsequent activation. Once activated, RIG-I/MDA5 bind to MAVS, which induces its dimerization and the recruitment of several molecules, including TOM70 and heat shock protein 90 (HSP90), to transduce downstream signalling, including TRAF6, TRAF5 and TBK1 [70,71]. This culminates in the translocation of the transcription factors IRF3, IRF7 and NF*κ*B to the nucleus and the subsequent expression of IFN*β* and pro-inflammatory cytokines. Interestingly, oxidative stress contributes to MAVS-mediated signalling since mtROS have been shown to induce MAVS oligomerization and type I IFN production [72].

Mitochondrial dynamics also impacts MAVS-mediated antiviral immune signalling since Drp1 or Fis1 knockdown, which both promote mitochondrial elongation, enhances MAVS-mediated signalling in response to Sendai virus infection and poly(I:C). Conversely, cells that have lost the ability to undergo mitochondrial fusion due to OPA1 or Mfn1 silencing present with impaired NFκB and IRF3 activation in response to the same conditions [65,73]. It is thought that by promoting an increase in ER–mitochondria contacts during viral infection, mitochondrial fusion enhances MAVS interactions with the stimulator of interferon genes (STING), found in the ER, resulting in activation of cGAS-STING signalling, which contributes to IFN production [65,74].

Viruses have developed several strategies that target mitochondria to antagonize innate immune signalling pathways and evade host immune defences [75,76]. The main strategies reviewed below include (i) mitochondrial fission and/or mitophagy triggering to reduce MAVS-dependent signalling and/or mitochondrial mass, respectively, (ii) disruption of ER–mitochondria contacts and (iii) directly targeting MAVS to prevent further downstream activation.

**Fig. 2.**
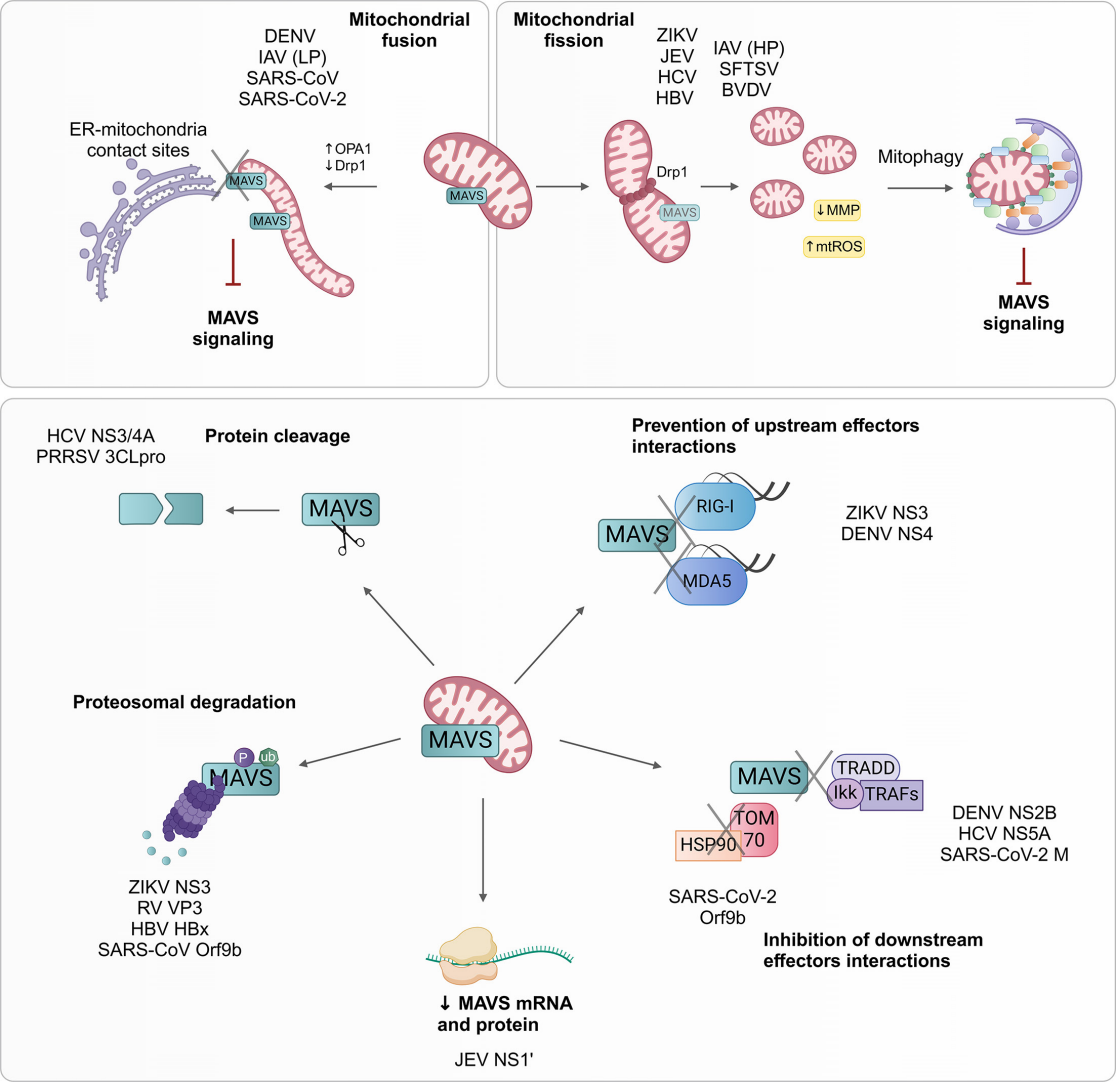
Modulation of mitochondrial dynamics and antiviral signalling during viral infection. By altering mitochondrial dynamics, viruses disrupt antiviral immune signalling to favour viral replication. Top left: several viruses promote mitochondrial fusion, which results in aberrant elongated mitochondria and disruption of ER contact sites, leading to inhibition of MAVS-mediated antiviral response. Top right: other viruses induce mitochondrial fragmentation through Drp1, which precedes mitophagy. Virus-induced mitophagy reduces mitochondrial mass and MAVS, resulting in blocking antiviral response. Bottom: viruses have also evolved mechanisms to directly inhibit MAVS, including protein cleavage and degradation, reduction of mRNA and protein levels, as well as inhibition of interactions of both upstream and downstream MAVS effectors. (LP: lowly pathogenic; HP: highly pathogenic). Figure made with BioRender.

Virus-induced mitochondrial fission and mitophagy are closely associated with the attenuation of innate immune response, given these processes can result in disruption of MAVS platform activation and, thus, suppression of the signalling cascade. For instance, inhibition of ZIKV-induced mitophagy by BNIP3 or NIX silencing in placental cells results in increased type I IFN response, thus impairing viral replication [34]. ZIKV NS4A reduces mitochondrial mass, MAVS oligomerization and hence IFN response, potentially via mitophagy induction, although direct interaction with MAVS cannot be dismissed [34]. Similarly, Drp1 silencing in HCV-infected Huh7 cells abrogates luciferase activity under the transcriptional control of IFN-stimulated response element [45], suggesting that mitochondrial fission regulates innate immunity. Recently, the nucleoprotein of the RNA bunyavirus severe fever with thrombocytopenia syndrome virus, a tick-borne ssRNA virus from the *Phenuiviridae* family, has been shown to inhibit MAVS-mediated signalling in HEK293T cells by inducing mitophagy and triggering MAVS degradation, favouring viral replication [77]. Moreover, influenza nucleoprotein-induced mitophagy attenuates innate immune response in HEK293T cells by degrading MAVS [58], while IAV PB1-F2, which localizes to mitochondria and promotes mitophagy, also inhibits RIG-I signalling due to MMP disruption and MAVS degradation [53,54]. Other examples of inhibition of IFN production via mitophagy include SARS-CoV-2 ORF10 [73,78] and bovine viral diarrhoea virus, another member of the *Flaviviridae* family [79] (Fig. 2). Therefore, viruses evade innate antiviral immunity by inducing mitophagy and interfering with MAVS-mediated signalling and, thereby, mitigating host antiviral defences.

However, virus-induced mitochondrial elongation during DENV, IAV H1N1 (low pathogenic strain) and SARS-CoV-2 infection is associated with disruption of ER–mitochondria contact sites and, in turn, reduced RIG-I signalling [40] (Fig. 2). Moreover, recovery of mitochondrial morphology and contacts with ER by Mito-C treatment during viral infection results in induction of antiviral innate signalling and inhibition of viral replication [41,59]. Therefore, viruses modulate mitochondrial dynamics and disrupt ER–mitochondria association to suppress RIG-I signalling and escape host innate immunity.

Viruses can also inhibit antiviral signalling by directly targeting specific proteins involved in RIG-I-like receptors (RLRs) signalling. Multiple mechanisms of RLR antagonisms employed by viruses have been described (reviewed in [75]); here only strategies involving mitochondria will be reviewed (Fig. 2). Given MAVS’s central role in the activation of antiviral immunity, many viruses target MAVS as a mechanism to reduce IFN production. Several viruses trigger MAVS degradation or inactivation. For instance, ZIKV NS3 protease binds to MAVS and promotes its ubiquitination and subsequent proteasome degradation, blocking IFN production [80]. DENV NS2B interacts with MAVS and IKK*ε* and blocks downstream IRF3 nuclear translocation and IFN production in HEK293T cells [81]. Likewise, ORF9b from SARS-CoV interacts with MAVS in A549 cells and promotes its ubiquitination, triggering MAVS proteasomal degradation via activating poly(rc) binding protein 2 as well as MAVS-interacting partners including TRAF3 and TRAF6 [60,82]. SARS-CoV-2 ORF9b binds to TOM70, impairing HSP90/TOM70 complex formation and the following IFN production [83,86], which may contribute to SARS-CoV-2 evasion of host innate immunity. HCV NS3/4A protease localizes to mitochondria and cleaves MAVS at Cys508 in HEK293T cells, causing the disassociation of the N-terminal domain and inactivating MAVS [87,88]. Moreover, HBV HBx protein interacts with MAVS and stimulates its ubiquitination and degradation via an unknown E3 ubiquitin ligase [89]. Similarly, VP3 protein from rotavirus, a dsRNA virus in the *Reoviridae* family, mediates MAVS phosphorylation, activating the proteasome pathway and promoting MAVS degradation in intestinal epithelial cells [90]. Porcine reproductive and respiratory syndrome virus (PRRSV), an ssRNA virus belonging to the *Arteriviridae* family, expresses a 3C-like serine protease (3CLpro) that cleaves MAVS and inhibits antiviral signalling in monkey epithelial cells [91]. Alternatively, JEV NS1 extended isoform (NS1′) suppresses both MAVS mRNA and protein levels and, thereby, IFN production by upregulating the expression of a microRNA (miR-22) in HeLa cells [92]. Other MAVS targeting strategies involve blocking the interaction of MAVS with active forms of RIG-I and MDA-5 to prevent complex formation and MAVS activation. For example, DENV NS4A localizes to the ER–mitochondria contact sites and interacts with MAVS, preventing MAVS’s subsequent interaction with RIG-I [93]. Likewise, ZIKV NS3 prevents RIG-I and MDA-5 mitochondrial translocation in HeLa cells by interacting with the adaptor proteins 14-3-3*ϵ* and 14-3-3*η*, respectively, thereby suppressing MAVS-mediated antiviral signalling [94]. SARS-CoV-2 glycoprotein M antagonizes antiviral signalling by interacting with MAVS in HEK293T cells, which impairs MAVS aggregation and the recruitment of downstream components [95]. In addition, HCV NS5A binds to MAVS and prevents the interaction of the following TRAF3 and TRAF6 proteins, blocking MAVS-mediated signalling [96]. Therefore, viruses have evolved many strategies to prevent MAVS activation and, yet more, distinct viral proteins from a virus target MAVS in different ways, ensuring inhibition of antiviral signalling throughout the viral cycle.

## Regulation of mitochondrial apoptotic pathway by viruses

Mitochondria are crucial in cell fate regulation not only because they provide energy to the cells but also because they mediate activation of the intrinsic apoptotic pathway. Upon mitochondrial damage, mitochondrial outer membrane permeabilization (MOMP) allows the release of mitochondrial proteins, in particular cytochrome c [97]. Binding of cytochrome c to apoptotic peptidase activating factor 1 results in the formation of the apoptosome and activation of the initiator caspase 9 and the executioner caspases 3 and 7 [98]. MOMP is mediated by members of the BCL-2 protein family, BAX and BAK proteins. During apoptosis, BAX/BAK accumulate at the OMM, and upon activation, they form higher-order multimers and lipid pores within the OMM, promoting MOMP [99,100]. In healthy cells, anti-apoptotic BCL-2 proteins prevent MOMP and the activation of the intrinsic pathway by inhibiting BAX and BAK [101].

The complex interplay between viruses and apoptosis has been extensively studied over the years and well-summarized previously [102]. Host cells activate apoptosis as a host defence mechanism to clear infection. Early induction of cell death by the host can impede viral protein and progeny production; thus, many viruses have evolved strategies to inhibit apoptosis. Once virions are mature, activation of cell death, either indirectly due to accumulated cellular damage or directly by upregulating apoptotic pathways, can be beneficial for some viruses, allowing release of viral progeny for their dissemination. However, other viruses suppress cell death throughout their cycle to promote a persistent infection. Therefore, regulation of apoptosis during viral infection is an intricate process, involving the integration of host defences and viral modulation strategies. This section will focus specifically on viral manipulation of the mitochondrial apoptotic pathway.

**Fig. 3.**
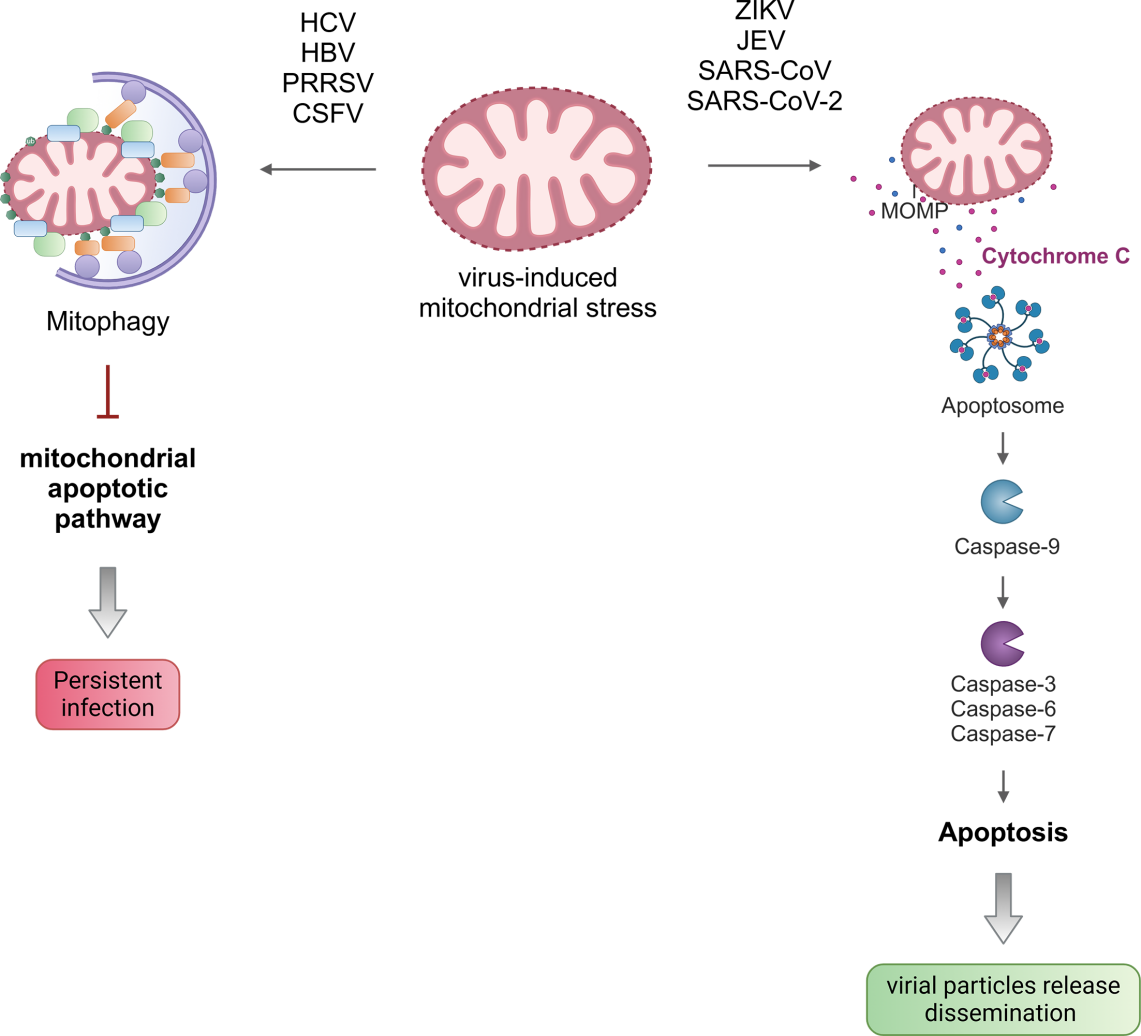
Modulation of mitochondria-mediated apoptosis during viral infection. Some viruses promote mitophagy to prevent further mitochondrial damage and activation of the mitochondrial apoptotic pathway. This allows them to establish a persistent infection. However, other viruses induce the intrinsic apoptotic pathway, which involves the release of cytochrome C from damaged mitochondria and the activation of the apoptosome and caspases, resulting in apoptosis. Virus-induced apoptosis allows the release of viral particles and viral dissemination. Figure made with BioRender.

Disruption of mitochondrial dynamics and function and increased mtROS levels can all lead to mitochondrial damage and release of pro-apoptotic proteins, ultimately resulting in induction of cell death. Viruses can inhibit mitochondrial-mediated cell apoptosis by activating mitochondrial quality controls such as mitophagy to remove damaged mitochondria, thus preventing activation of the intrinsic pathway and ensuring their replication. For instance, inhibition of HCV-induced mitophagy in Huh7 cells results in increased cytosolic cytochrome C levels, caspase 3 activity and thus, cell death, indicating that HCV-mediated mitophagy attenuates cell apoptosis and facilitates persistent viral replication [45]. Similar results were found in HBV-infected Huh7 cells. Disruption of Parkin-mediated mitophagy leads to cytochrome c release and activation of caspase 3, resulting in cell death [49]. Likewise, PRRSV and classical swine fever virus (CSFV), an ssRNA belonging to the *Flaviviridae* family, induce mitochondrial fission via Drp1 and Parkin/PINK1-mediated mitophagy [91,103]. Interference of mitochondrial fission and mitophagy by Drp1 and Parkin silencing, respectively, in both PRRSV- and CSFV-infected cells enhances cytochrome c release and caspase 3-mediated apoptosis while reducing viral replication (Fig. 3). Altogether, virus-induced mitophagy attenuates apoptosis to allow a persistent viral infection.

In contrast, viruses including ZIKV, JEV, SARS-CoV and SARS-CoV-2 can induce cell apoptosis to promote viral release and enhance spreading and dissemination. Yang *et al.* found that ZIKV-induced mitochondrial fragmentation precedes the activation of the mitochondrial intrinsic apoptotic pathway in human neural stem cells [35]. Of note, mitophagy was not monitored in these cells. Interestingly, restriction of mitochondrial fragmentation by chemically inhibiting Drp1 leads to an increase in cell survival after ZIKV infection of neuronal cells. Similarly, JEV infection promotes mitochondrial stress and caspase 3-mediated apoptosis in different cells, including human neuroblastoma [104] and medulloblastoma cell lines [105]. Specifically, NS2B-NS3 protease expression induces ROS production, disruption of MMP and mitochondrial cell death [105]. Several SARS-CoV proteins have been associated with the induction of the mitochondrial apoptotic pathway. First, ORF3a expression results in cytochrome c release and BAX-mediated apoptosis activation [106]. Second, the nucleocapsid protein also induces the apoptotic pathway during starvation conditions by depolarizing mitochondria, increasing ROS production and triggering cytochrome c release to the cytosol [107]. Finally, membrane protein has been shown to promote the release of cytochrome c and induce cell apoptosis [108]. Similarly, membrane protein from SARS-CoV-2 induces caspase-associated apoptosis via activating the pro-apoptotic BOK, which aggravates lung damage *in vivo* [109]. In agreement with this, SARS-CoV-2 infection promotes mitochondria dysfunction and caspase 3-mediated apoptosis in human pluripotent stem cell-derived cardiomyocyte-like cells [110]. Surprisingly, SARS-CoV-2 nucleocapsid protein inhibits cell apoptosis via promoting anti-apoptotic function of MCL-1 [111] (Fig. 3). These findings suggest that distinct viral factors can exert anti- or pro-viral roles and that their expression during the early/late phases of infection will determine the global outcome. Although suppression of the intrinsic pathway occurs during HCV infection, expression of HCV viral proteins alone triggers apoptosis. HCV NS4A and NS3-NS4A expression in HEK293T and Huh7 cells induces alterations in MMP as well as high mtROS production, resulting in mitochondrial damage and the release of cytochrome c into the cytoplasm [112,113]. NS4A mediates activation of the mitochondrial pathway by upregulating the pro-apoptotic protein Bax. NS4B was also shown to induce cell death through the mitochondrial apoptotic pathway [114]. However, some of the effects reported here might be altered in the context of infection given the high expression level and lack of differential regulation that other viral proteins may contribute to. Altogether, viruses have evolved different strategies that converge in a complex signalling network that regulates cell death depending on viral demands during different stages of the virus cycle, as well as tissue specificity.

While the mechanisms regulating apoptosis and mitochondrial autophagy (mitophagy) are not fully understood, evidence suggests that a tight spatiotemporal control of these processes is crucial to ensure a balanced state during the different stages of the viral cycle. Rotavirus and IAV are clear examples of how apoptosis is differently modulated at different times of the viral cycle [115]. During early rotavirus infection of Vero and MA104 cells, NSP1 interacts with p53 and mediates its proteasomal degradation, inhibiting apoptotic initiation [116]. p53 expression is then increased at later stages and it partially mediates rotavirus-induced apoptosis. Moreover, a recent paper found that rotavirus NSP4 induces mitochondrial fragmentation in a Drp1-dependent manner at later stages of infection in MA104 cells, promoting release of cytochrome c to the cytosol and activation of caspase 3. Inhibition of mitochondrial fission results in attenuation of apoptotic markers as well as viral release [117]. In this manner, rotavirus prevents cellular apoptosis at early stages to allow sufficient time for replication, viral protein production and virion assembly. Then, cell apoptosis is induced to promote the release of virions from the infected cells and allow viral dissemination at later stages.

Overall, it is tempting to speculate that almost all viruses suppress host-induced cell death at early stages to promote viral replication and virion formation. Then, depending on the outcome of the infection – lytic or latent – some viruses induce cell apoptosis to permit viral shedding and spreading, while others continue inhibiting apoptosis to allow persistent infection. In addition, virus interactions with mitochondria may also lead to or regulate other cell death pathways such as ferroptosis, pyroptosis and necroptosis.

## Regulation of mitochondrial oxidative and antioxidant responses during viral infection

Mitochondria are one of the main sources of ROS from the respiratory chain [118,119]. Electrons in the ETC leak out from the complexes and interact with oxygen to produce superoxide or hydrogen peroxide [120]. Suppression of electron transfer from complexes I and III to subsequent electron carriers by the inhibitor rotenone and antimycin A, respectively, results in electron accumulation and, potentially, electron leakage and superoxide generation [121,122]. Several factors, including substrate availability, rate of ATP production or MMP, differently alter ETC complexes, contributing to ROS production [123]. ROS are highly reactive, and excessive ROS levels can induce oxidative stress and damage DNA and proteins, causing mitochondrial dysfunction and cell death [124]. Therefore, mitochondria exploit the antioxidant systems to control the amount of ROS produced and alleviate this stress. Superoxide dismutases (SODs), and specifically SOD2 in the mitochondria, constitute an important antioxidant defence system converting superoxide into a less reactive molecule, H_2_O_2_ [125]. H_2_O_2_ can then be further catalysed into water by the glutathione and thioredoxin/peroxiredoxin systems [126,127].

Beyond toxicity, evidence suggests that ROS can act as a second messenger to regulate intracellular signalling. Specifically, ROS oxidize metal cofactors or cysteine residues from different cytosolic proteins, changing their function. It is well established that mtROS can contribute to hypoxic adaptation, cell proliferation and differentiation, innate immune response, regulation of phosphorylation signalling, control of gene expression and apoptosis [128,132]. The amount of ROS produced within the cells, together with the scavenger activity of antioxidant systems, determines whether ROS function as beneficial or detrimental.

Alteration of mitochondrial respiration and dynamics is one of the main triggers of mtROS production. Since viruses greatly modulate mitochondrial dynamics, it is not surprising that viral infection results in elevated levels of mtROS. Although it is not fully well understood whether certain ROS in the mitochondria or elsewhere are pro-viral or antiviral, multiple studies have demonstrated that inhibition of virus-induced ROS through antioxidant treatment suppresses viral replication, suggesting that ROS production during viral infection is beneficial for viruses.

By disrupting mitochondrial dynamics, mitochondrial respiration and MMP, ZIKV infection induces high mtROS levels in both placental and iPSC-derived astrocytes [34,133]. Similarly, DENV-induced MMP disruption in human brain endothelial cells is associated with mtROS accumulation [134]. Interestingly, ROS inhibition with various antioxidants, including *N*-acetylcysteine (NAC) and mitoTEMPO, decreases DENV-induced cell death, partially restores endothelial permeability and significantly reduces viral replication and production of infectious particles. These findings demonstrate a pro-viral effect of elevated ROS during DENV infection, which may be involved in its pathogenesis. Likewise, respiratory syncytial virus (RSV) infection, a negative-sense ssRNA virus from the *Pneumoviridae* family, results in compromised mitochondrial respiration, MMP and increased mtROS levels in A549 and human embryonic kidney cells [135,136]. RSV-induced mtROS are essential for viral replication and virion production, as treatment with mitochondria ROS scavengers significantly reduces viral titres. In agreement with these results, an independent study also reported that RSV infection, as well as influenza infection, promotes increased levels of ROS in A549 cells [137]. NAC treatment induces a reduction in ROS levels, which leads to a decrease in mucin synthesis, pro-inflammatory cytokines expression and viral titres, suggesting that certain ROS favour viral replication and contribute to viral-associated pathogenesis.

Furthermore, virus-induced endoplasmic reticulum (ER) stress can promote mtROS production. Stress in the ER induces Ca^2+^ efflux from ER to mitochondria, which can result in higher mitochondrial ROS production [138]. Additionally, ER stress disrupts ER–mitochondria contact sites, altering mitochondrial dynamics and contributing to ROS generation. For instance, HCV core protein promotes mtROS production by increasing Ca^2+^ entry rate from the ER due to stimulation of the mitochondrial calcium uniporter complex activity [139]. In addition, HCV NS5A-induced ROS are essential factors in modulating mitophagy induction, as NAC treatment abolished NS5A-induced mitophagy [47]. However, how ROS drive NS5A-induced mitophagy, as well as their implication in disease progression, remains unknown.

Although the molecular mechanisms by which high ROS levels may be beneficial for viruses are not fully understood, it might be due to (i) a direct mechanism by which oxidation of viral proteins modulates their activity and (ii) an indirect mechanism by which ROS regulates cellular signalling pathways or (iii) the effects of a specific ROS (e.g. hydrogen peroxide) during viral infection. ROS can induce oxidation of intracellular proteins, regulating their function. For instance, oxidative conditions are crucial for proper SARS-CoV-2 entry into host cells [140]. SARS-CoV-2 spike protein interacts with host receptor angiotensin-converting enzyme-2 (ACE2) to mediate viral attachment and cell fusion. Four disulphide bonds found within the spike’s RNA-binding domain are needed for membrane fusion. Consistently, disruption of these disulphide bonds by treatment with reducing agents inhibits cell fusion and viral entry in ACE2-expressing HEK293T cells. Furthermore, the guanylyltransferase activity of the NS5 RNA capping enzyme from members of the *Flaviviridae* family is enhanced under oxidative conditions [141]. Accordingly, antioxidant treatment results in reduced viral replication in baby hamster kidney (BHK) cells, indicating that flaviviruses exploit oxidative stress to control RNA capping and genome synthesis. Virus-induced ROS can also modulate cellular pathways for its own benefit. Infection with an avian coronavirus, infectious bronchitis virus (IBV), promotes ROS production, which is associated with loss of MMP in chicken macrophages [142]. Reduction of IBV-induced ROS levels with antioxidants inhibits mitochondrial-mediated apoptosis and reduces viral titres, suggesting that increased ROS levels are important to mediate cell death, enhancing viral shedding [142].

In other situations, ROS production might be exploited by the host cell to prevent viral infection. HSV-1 completes a fully permissive infection in epithelial cells and neurons, while it results in a non-productive infection in mononuclear cells of the innate immunity. Marino-Merlo *et al.* showed that HSV-1 infection of monocytes results in an early burst of ROS production driven by NFκB activation, which plays a central role in restricting viral replication in monocytic cells [143]. Consequently, selenium-based antioxidant treatment abolishes HSV-1-induced ROS, leading to increased viral replication in an NFκB-dependent manner. This suggests that monocytes induce NFκB-mediated ROS production to suppress viral replication, potentially by inducing an antiviral state. Therefore, ROS production is detrimental for HSV-1 and contributes to limiting viral infection in mononuclear immune cells.

Uncontrolled ROS levels may also result in adverse outcomes for both viruses and host cells. Thus, antioxidant defence mechanisms can be activated to counteract excessive ROS production. Infection of hamster kidney fibroblast with the flavivirus West Nile virus (WNV) induces both high ROS production and increased glutathione (GSH) levels to counterbalance the oxidative stress [144]. In addition, the transcription factor nuclear factor erythroid 2-related factor 2 (Nrf2), which drives expression of antioxidant genes, is activated during WNV infection. Although viral titres are not significantly altered by ATF4 or Nrf2 silencing, inhibition of antioxidant defences leads to a decrease in viral NS3 protein levels. This implies that WNV-induced ROS are counteracted by high GSH levels, maintaining a balanced redox state which may provide benefits for the virus by preventing excessive oxidative damage. In a similar way, SARS-CoV-2 infection promotes mtROS production in Vero and Huh7 cells as well as upregulation of antioxidant defences including glutathione-S-transferase (GSTP1) [62,145, 146]. Tang *et al.* recently discovered that piperlongumine, an alkaloid compound extracted from long pepper, selectively inhibits the overexpressed antioxidant enzyme GSTP1 in both Vero and A549 infected cells, promoting excessive ROS levels which reduce SARS-CoV-2 replication *in vitro* and delay disease progression in a mouse model [147]. Similarly, infection of human fibroblasts with human cytomegalovirus (HCMV), a DNA virus member of the *Herpesviridae* family, induces early ROS production which is rapidly neutralized by upregulation of GSH and antioxidant enzymes including SOD and GPX1 [148,149]. Interestingly, the HMCV-induced antioxidant state is required for the production of viral progeny and maintenance of mTORC1 activity, which is inhibited under oxidative stress conditions. Therefore, a fine balance of the redox state, regulated by antioxidant cellular response, is essential for infection progression.

In contrast, DENV counteracts increased ROS levels in A549 and primary human dendritic cells by targeting Nrf2 for NS2B3-mediated degradation, thereby inhibiting antioxidative defences [150]. This results in increased oxidative stress, which contributes to enhanced viral replication and inflammation. Similarly, HCV NS3 also interferes with host antioxidant response by binding to Nrf2 and preventing its nuclear translocation and subsequent activation [151]. Although this inhibition does not directly enhance viral replication, the HCV-induced redox imbalance may be associated with its pathogenesis. Furthermore, JEV also impairs antioxidant defences to maintain increased high ROS production in human promonocyte cells [152]. JEV infection induces ROS production and downregulates thioredoxin levels, resulting in mitochondrial-mediated apoptosis. In agreement with these findings, Singh *et al.* reported increased ROS levels, NOX2 upregulation and low levels of GSH at later stages of JEV-infected mice brain cortex [38,152]. Therefore, these findings indicate that viruses have evolved context-dependent sophisticated strategies to co-opt cellular redox balance to favour viral replication and infection progression. Given the crucial role of redox homeostasis during viral infection, future studies are required to determine whether drugs altering cellular redox status may be used in the treatment or control of viral diseases.

## Regulation of the mitochondrial unfolded protein response signalling by viruses

While mitochondria contain their own genetic material (mtDNA), most mitochondrial proteins are encoded by nuclear genes [153]. These nuclear-encoded proteins are synthesized in the cytosol and imported into mitochondria where they are folded and processed by mitochondrial chaperones and proteases. Mitochondrial stress can impair mitochondrial protein homeostasis (proteostasis), resulting in increased unfolded proteins and triggering mitochondrial dysfunction, ultimately affecting other cellular processes [154]. Therefore, cells have evolved tailored quality control processes that sense and resolve mitochondrial proteostasis to restore mitochondrial homeostasis. The mitochondrial unfolded protein response (UPR^mt^) consists of a protective transcriptional response mediated by mitochondrial-nuclear communication whose aim is to increase mitochondrial folding capacity through the induction of the mitochondrial chaperones including Hsp10, Hsp60 and Hsp90 and proteases such as ClpP and LONP1 [155]. UPR^mt^ is engaged upon a wide variety of stressors, including an accumulation of misfolded and/or truncated proteins within the mitochondrial matrix, mitonuclear protein imbalance, disruption of mitochondrial protein import, mtDNA depletion, reduction of mitochondrial protein synthesis, elevated mtROS levels, impairment of OXPHOS and citric acid cycle and dissipation of MMP [155,161]. UPR^mt^ signalling is primarily mediated by activating transcription factor 5 (ATF5) and heat shock transcription factor 1 [162,163]. An activated UPR^mt^ appears to be crucial to enhance mitochondrial folding capacity and induce a mitochondrial recovery programme, alleviating neurodegenerative and cardiovascular disease symptomatology [164,165], promoting resistance to pathogen infection [166,167] and ageing [168].

Since multiple viruses affect and exploit mitochondrial function during their cycle, UPR^mt^ activation can act either as a host defence mechanism or viral strategy to maintain viral infection. Albeit little work has investigated specifically the role of the UPR^mt^, few studies have reported UPR^mt^ activation and its consequences during viral infection. For instance, expression of fusion and haemagglutinin-neuraminidase (HN) glycoproteins from Newcastle disease virus (NDV), a negative-sense ssRNA virus belonging to the *Paramyxoviridae* family, induces syncytium formation and membrane fusion in A549 cells [169]. Interestingly, fusion and HN co-expression do not damage mitochondria nor induce mitophagy but also promote mitochondrial biogenesis by activating the UPR^mt^. Therefore, activation of UPR^mt^ maintains mitochondrial integrity and ensures proper energy supply during syncytium formation. Future studies should aim at elucidating whether preventing UPR^mt^ activation influences syncytium formation and/or NDV infection. Moreover, ATF5 translational upregulation was also shown to occur during viral infection of murine cells with the betacoronavirus mouse hepatitis virus [170]. Likewise, ectromelia virus (ECTV), an enveloped DNA virus from the *Poxviridae* family, induces a mitochondrial heat shock response by inducing HSPD1 and HSPE1 protein levels in murine fibroblasts [171]. ECTV infection leads to increased levels of anti-apoptotic proteins and reduced levels of pro-apoptotic Bax protein, indicating a decrease in apoptotic potential in fibroblasts upon ECTV infection. Therefore, these findings suggest that activation of UPR^mt^ maintains mitochondrial proteostasis and prevents activation of host apoptotic pathways, ensuring survival of infected cells. Similarly, infection of glioma cells with HCMV induces ATF5 expression at both protein and RNA levels as well as activation of the cellular stress response [172]. Interestingly, HCMV infection sustains high ATF5 levels by downregulating a microRNA, miR-134-5p, which negatively regulates ATF5 transcription. It was hypothesized that ATF5 upregulation provides resistance to apoptosis, potentially by maintaining mitochondria homeostasis and allowing a life-long persistent infection, although further experiments, including how ATF5 silencing affects cell viability and viral infection, are required to verify that.

Chronic HBV infection can result in fibrosis accumulation – defined by fibrous scar accumulation as the liver is constantly repairing itself due to chronic inflammation – and eventually cirrhosis, which is highly associated with liver failure and liver cancer development [173]. Recently, it was shown that chronic HBV patients exhibit mtDNA deletions and reduced mtDNA copy number potentially due to enhanced oxidative stress and mitochondrial dysfunction [174]. Interestingly, Loureiro *et al*. found that chronic HBV patients with mild fibrosis present increased mRNA levels of UPR^mt^ genes, including the mitochondrial protease LONP1 and the mitochondrial chaperones HSPA9 and HSPD1, compared to patients with advanced fibrosis. Moreover, other mitochondrial protective mechanisms, including mitophagy and antioxidant enzymes, are also increased in chronic HBV patients with mild fibrosis compared to those with severe fibrosis. These findings provide evidence that activation of protective mitochondrial mechanisms such as UPR^mt^, mitophagy and antioxidant defences maintains mitochondrial integrity during fibrosis, preventing its further development. In contrast, impairment or downregulation of these mitochondrial adaptive mechanisms is associated with severe stages of fibrosis and cirrhosis. Further work is needed to shed light on the therapeutic potential of boosting adaptive mitochondrial mechanisms to improve fibrosis symptomatology and impair further fibrosis development.

## Outstanding questions and perspectives

**Fig. 4.**
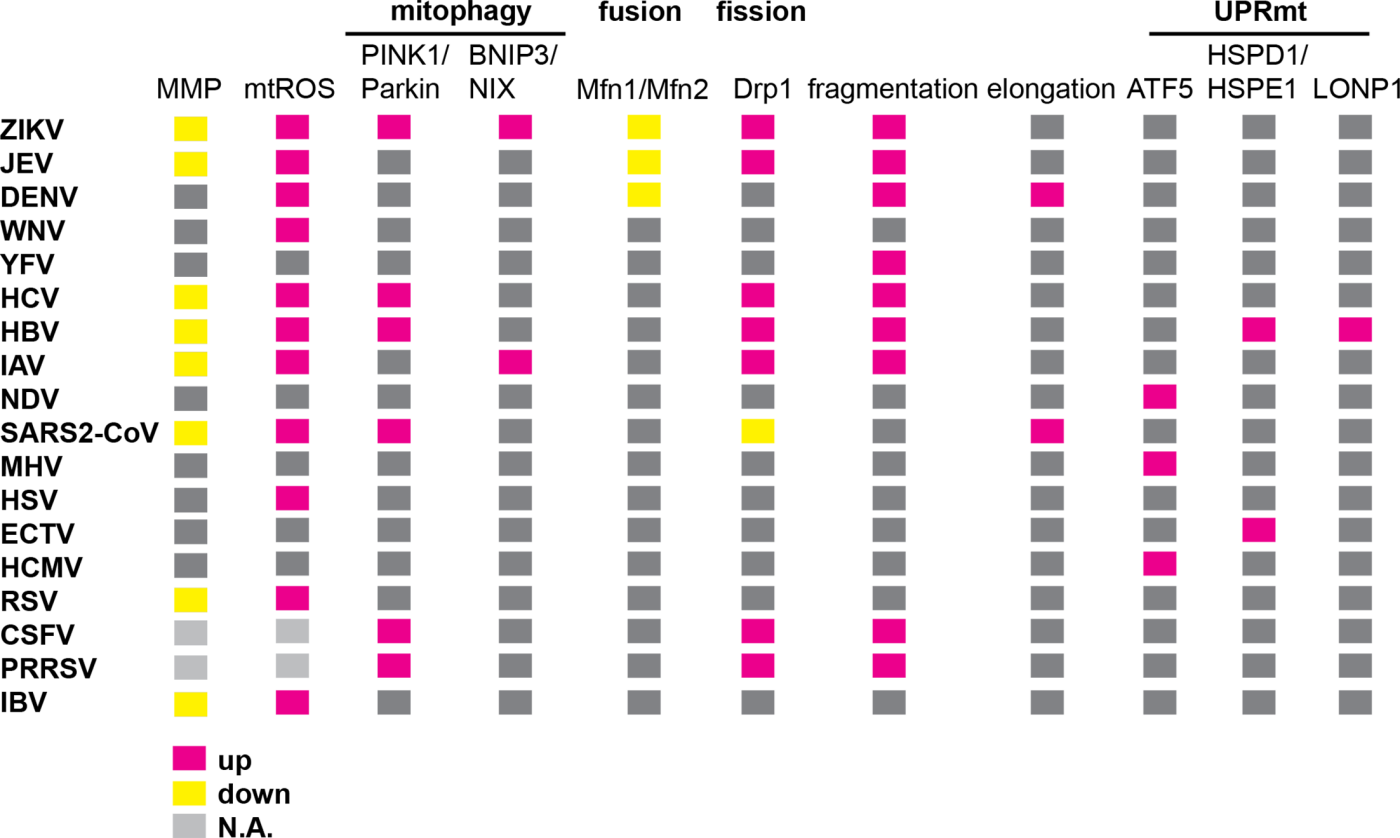
Summary of mitochondrial perturbations during viral infections. Alterations of individual mitochondrial functions for individual viruses are grouped and displayed either as upregulated (red), downregulated (yellow) or unknown/not available (grey).

The multifaceted contributions of mitochondria to cellular functions, including antiviral defence, apoptosis and signalling, make them critical targets during viral infections. From this review, it is apparent that some viruses promote mitochondrial homeostasis by activating protective signalling cascades and responses – such as mitophagy, antioxidant systems and the UPR^mt^ – to prevent cell death and allow a persistent infection. In contrast, other viruses hijack mitochondria for their own benefit, disrupting mitochondrial integrity and inducing cell death, which is associated with their pathogenesis. These virus-specific differential regulations of mitochondrial function are all summarized in Fig. 4. Although significant research has explored how mitochondria are repurposed by viruses, many issues remain unsolved. First, while several proteins directly target mitochondria to modulate dynamics and function, the exact mechanisms through which other viral proteins regulate mitochondrial activity, e.g. by interacting with host factors, are not fully clear. Furthermore, questions remain regarding the molecular mechanisms underlying viral strategies that shift mitochondrial dynamics towards fragmentation or elongation. Experiments with primary cells and diverse viral strains may help us understand how mitochondrial dynamics impact viral pathogenicity. Second, in cases where viral proteins exert opposing effects on mitochondrial dynamics, apoptosis and redox balance, investigating the spatiotemporal regulation of these mechanisms during the course of the infection may provide insights into how mitochondrial modulation influences the balance between viral replication versus antiviral signalling. In addition, further research is needed to elucidate the precise molecular mechanisms that determine whether virus-induced ROS act in a pro-viral or antiviral manner and how they modulate host cell signalling. Third, the interplay among mitochondrial quality control processes such as mitophagy, UPR^mt^ and antioxidative defences remains largely unexplored. Further research is needed to identify the signals that drive the shift between cell survival and cell death pathways, as well as whether activation of these quality control processes facilitates virus replication or serves as host protective mechanisms. Fourth, validating these findings in animal models will shed light on how regulation of mitochondrial function contributes to pathogenesis and the development of viral diseases. Finally, future work should also address whether targeting mitochondrial pathways could be considered as a therapeutic strategy for the development of broad-spectrum antivirals and the potential adverse effects on host cellular functions.

## Abbreviations

CSFV: classical swine fever virus
DENV: dengue virus
ECTV: ectromelia virus
ER: endoplasmic reticulum
ETC: electron transport chain
GSH: glutathione
HBV: hepatitis B virus
HCMV: human cytomegalovirus
HCV: hepatitis C virus
HN: haemagglutinin-neuraminidase
IAV: influenza A virus
IBV: infectious bronchitis virus
IMM: inner mitochondrial membrane
JEV: Japanese encephalitis virus
MAVS: mitochondrial antiviral signalling protein
MMP: mitochondrial membrane potential
MOMP: mitochondrial outer membrane permeabilization
mtROS: mitochondrial reactive oxygen species
NAC: *N*-acetylcysteine
NDV: Newcastle disease virus
OMM: outer mitochondrial membrane
OXPHOS: oxidative phosphorylation
PRRSV: porcine reproductive and respiratory syndrome virus
RLR: RIG-I-like receptor
ROS: reactive oxygen species
RSV: respiratory syncytial virus
SARS-CoV: severe acute respiratory syndrome coronavirus
SOD: superoxide dismutase
UPR^mt^: mitochondrial unfolded protein response
WNV: West Nile virus
YFV: yellow fever virus
ZIKV: Zika virus
